# CD14 −159 C>T Gene Polymorphism with Increased Risk of Tuberculosis: Evidence from a Meta-Analysis

**DOI:** 10.1371/journal.pone.0064747

**Published:** 2013-05-31

**Authors:** MY. Areeshi, Raju K. Mandal, Aditya K. Panda, Shekhar C. Bisht, Shafiul Haque

**Affiliations:** 1 Department of Medical Microbiology, College of Nursing and Allied Health Sciences, Jazan University, Jazan, Saudi Arabia; 2 Department of Urology, Sanjay Gandhi Post Graduate Institute of Medical Sciences, Lucknow, Uttar Pradesh, India; 3 Department of Infectious Disease Biology, Institute of Life Sciences, Bhubaneswar, Odisha, India; 4 Department of Biotechnology, Hemwati Nanadan Bahuguna Garhwal University, Srinagar (Garhwal), Uttarakhand, India; 5 Department of Biosciences, Jamia Millia Islamia (A Central University), New Delhi, India; Institute of Microbial Technology, India

## Abstract

Cluster of differentiation 14 (CD14) gene is an important component of the human innate immune system and its role in tuberculosis (TB) has been sparsely documented. The enhanced plasma CD14 levels in TB patients as compared to healthy controls are associated with CD14 gene promoter (C-159T) polymorphism. In the past few years, the relationship between CD14 −159 C>T (rs2569190) polymorphism and risk of TB has been reported in various ethnic populations; however, those studies have yielded contradictory results. In this study systemic assessment was done for the published studies based on the association between CD14 −159 C>T polymorphism and TB risk retrieved from PubMed (Medline) and EMBASE search. A total number of 1389 TB cases and 1421 controls were included in this study and meta-analysis was performed to elucidate the association between CD14 −159 C>T polymorphism and its susceptibility towards TB. Pooled odds ratios (ORs) and 95% confidence intervals (95% CIs) were calculated for allele contrast, homozygous comparison, heterozygous comparison, dominant and recessive genetic model. It was found that T allele carrier was significantly associated with increased TB risk (T vs. C: p-value = 0.023; OR = 1.305, 95% CI = 1.038 to 1.640). Similarly, homozygous mutant TT genotype also revealed 1.6 fold increased risk of TB (TT vs. CC; p-value = 0.040; OR = 1.652, 95% CI = 1.023 to 2.667). Additionally, dominant genetic model demonstrated increased risk of developing TB (TT vs. CC+CT: p-value = 0.006; OR = 1.585, 95% CI = 1.142 to 2.201). The study demonstrates that CD14 gene (−159 C>T) polymorphism contributes increased susceptibility for TB. Moreover, this meta-analysis also suggests for future larger studies with stratified case control population and biological characterization for validation studies.

## Introduction

Tuberculosis (TB) is an infectious disease, remains a major public health concern and leading cause of morbidity and mortality across the globe. According to the World Health Organization (WHO), approximately 2 million people die annually due to tuberculosis [1]. It is estimated that approximately one-third of the world’s population is infected with *Mycobacterium tuberculosis* (MTB), the causative agent of tuberculosis, among them only 10% develop clinical disease [2]. This has maintained interest in that there is an inter-individual heterogeneity in host susceptibility for TB. Increasing evidences suggested that genetic variants might contribute to the underlying pathophysiology of TB at the individual level [3], [4], although the exact mechanism is still unclear.

CD14 is a key pattern recognition receptor involved in the innate immune response and renowned co-receptor for numerous microbial products and other bacterial wall-derived components [5], [6]. CD14 has several other functions, including the clearance of apoptotic cells as it is constitutively expressed, primarily on the surface of monocytes, macrophages, and neutrophil [7], [8]. CD14 exists in both membrane-bound (mCD14) and soluble forms (sCD14). Soluble CD14 (sCD14) is present in the circulation and other body fluids, and its level increases in serum plasma during inflammation and other infectious diseases [9], [10]. Similar is the case of TB, where increased level of CD14 has been reported in TB patients [11].

A polymorphism C>T identified on the −159 position of promoter region of CD14 gene and found to be linked with increased transcriptional activity that affects expression level of CD14 [12]. Given the functional significance of this genetic variant, a number of case-control studies have been done in different populations to investigate its susceptibility towards TB, but the findings remain conflicting rather than conclusive [13]–[18]. Inconsistencies in results can be attributed in terms of sample size and ethnic diversity, and individual studies might lack sufficient strength and recognition to achieve a comprehensive and reliable conclusion. Meta-analysis is a significant tool for analyzing cumulative data from studies where individual sample sizes are small and represent lower statistical power [19], [20]. Hence, a quantitative synthesis may provide more clear evidence on the association of such genetic polymorphisms with TB. In view of above we conducted a meta-analysis study based on published research articles to make a more comprehensive and compelling evaluation of the connection between −159 C>T polymorphism and TB risk.

## Materials and Methods

### Identification and Eligibility of Relevant Studies

We performed a PubMed (Medline) and EMBASE search covering all research articles published with a combination of the following key words: ‘CD14 gene or CD14 polymorphisms’ and ‘Tuberculosis’ (last updated on February, 2013). We evaluated potentially relevant genetic association studies by examining their titles and abstracts, and all published studies matching with the eligible criteria were retrieved.

### Inclusion and Exclusion Criteria

In order to minimize heterogeneity and facilitate the appropriate interpretation and understanding of the findings, studies included in the current meta-analysis had to meet the following criteria: a) evaluation of CD14 −159 C>T polymorphism and TB risk, b) use of case-control design, c) recruitment of pathologically confirmed TB patients and TB free controls, d) have available genotype frequency in cases and controls, e) the articles must be published in English language. Among the retrieved articles, if any of the case-control study was included by more than one article using the same case series, we selected the study that included the largest number of individuals. The major reasons for exclusion of the studies were, [i] overlapping data, and [ii] case-reports and review articles.

### Data Extraction and Quality Assessment

For each research publication, the methodological quality assessment and data extraction was independently abstracted in duplicate by two independent investigators using a standard protocol and data-collection form according to the inclusion criteria listed above to ensure the accuracy and consistency of the data. In case of disagreement on any item of the data, the problem was fully discussed in order to reach a mutual consensus. Characteristics abstracted from the studies included the first author’s name, year of publication, country of origin, sources of cases and controls, number of cases and controls, type of study, and genotype frequencies.

### Statistical Analysis

Meta-analysis is a unique technique which merges outcomes of independent similar type of studies and derives a definitive conclusion for wider applications [19], [20]. The statistical analysis for the present study was done by using the (CMA) V2 meta-analysis comprehensive software (Biostat, USA). Meta-analysis program CMA V2 has some advantages over other programs currently available in a market for computing meta-analysis study (http://www.meta-analysis.com/pages/comparisons.html). We calculated the ORs and corresponding 95% CI values, to evaluate the association between CD14 −159 C>T polymorphism and TB risk. Basically, heterogeneity in meta-analysis refers to the variations in outcomes between different studies. Hence, heterogeneity assumption was checked by the chi-square based Q-test [21], p-value >0.05 for the Q-test indicated a lack of heterogeneity among the studies. The pooled ORs were calculated either by the fixed effects model (Mantel-Haenszel method) [22] or the random-effects model (Der Simonian and Laird) [23]. Additionally, I^2^ statistics was employed to quantify inter-study variability. It was in range of 0% to 100%, where a value of 0% indicated no observed heterogeneity, and higher values signified an increasing degree of heterogeneity [22]. Hardy-Weinberg equilibrium (HWE) in the control group was measured by chi-square test and significance level was maintained at p-value <0.05. Publication bias was examined through visual inspection of the funnel plots in which the standard error of log (OR) of each study had been plotted against its own log (OR), for i.e., an asymmetric plot indicated a possible publication bias [24], [25]. Funnel plot asymmetry was evaluated by Egger’s linear regression test, which is a linear regression approach for the measurement of funnel plot asymmetry on the natural logarithmic scale of the OR [20], [26]. The significance of the intercept was determined by the t-test (p-value <0.05 was considered as a representation of statistically significant publication bias) [19].

## Results

### Characteristics of Published Studies

A total number of forty (N = 40) research articles were retrieved through literature search from the PubMed (Medline) and EMBASE web portals/databases (Figure S1). All retrieved articles were examined by reading the titles and abstracts, and full texts for the potentially relevant publications were further checked for their suitability of incorporation in this meta-analysis. Besides the database search, the references listed in the retrieved articles were also screened for other potential articles. Studies either using CD14 polymorphism to predict survival in TB patients or considering CD14 variants as indicators for response to therapy were excluded. Moreover, studies based on investigation of the levels of CD14 mRNA or protein expression or review articles were also excluded. We included only case-control or cohort design studies showing frequency of all three genotypes. After careful screening and following the inclusion and exclusion criteria, we found seven (N = 7) eligible original published studies and included in this meta-analysis (Table 1). The distribution of genotypes in the controls did not deviate from HWE (Table 2).

**Table 1:** Main characteristics of all seven studies included in a meta-analysis.

Authors	Year	Country of origin	Study design	Genotyping method	Cases	Controls
Alavi-Naini et al., 2012 [13]	2012	Iran	HB	ARMS PCR	120	131
Ayaslioglu et al., 2012 [14]	2012	Turkey	HB	PCR-RFLP	88	116
Zhao et al., 2012 [15]	2012	China	HB	PCR-Sequencing	410	404
Kang et al., 2009 [16]	2009	Korea	HB	PCR-RFLP	274	422
Rosas-Taraco et al., 2007 [11]	2007	Mexico	HB	PCR-RFLP	104	114
Druszczyńska et al., 2006 [17]	2006	Poland	HB	PCR-RFLP	126	122
Pacheco et al., 2004 [18]	2004	USA	HB	PCR-RFLP	267	112

Note: HB = Hospital based.

**Table 2:** Distribution of gene polymorphism of studies included in a meta-analysis.

Authors, year and reference	Control				Case					TB risk
	Genotype			Minor allele	Genotype			Minor allele	HWE (p-value)	
	CC	CT	TT	MAF	CC	CT	TT	MAF		
Alavi-Naini et al., 2012 [13]	38	71	22	0.43	18	66	36	0.57	0.25	Significant association
Ayaslioglu et al., 2012 [14]	15	59	42	0.61	16	43	29	0.57	0.41	No association
Zhao et al., 2012 [15]	76	208	120	0.55	75	149	186	0.63	0.39	Significant association
Kang et al., 2009 [16]	72	215	135	0.57	39	118	117	0.64	0.38	Significant association
Rosas-Taraco et al., 2007 [11]	37	63	14	0.39	16	51	37	0.60	0.10	Significant association
Druszczyńska et al., 2006 [17]	48	59	19	0.38	38	59	25	0.44	0.90	No association
Pacheco et al., 2004 [18]	31	54	27	0.48	92	119	56	0.43	0.71	No association

### Publication Bias

Begg’s funnel plot and Egger’s linear regression test were performed to assess the publication bias in the reports included for meta-analysis [20], [26]. The shape of funnel plots and Egger’s test did not show any evidence of publication bias (Table 3).

**Table 3:** Statistics to test publication bias and heterogeneity in a meta-analysis.

Comparisons	Egger’s regression analysis			Heterogeneity analysis			Model used for meta-analysis
	Intercept	95% Confidence Interval	p-value	Q-value	P_heterogeneity_	I^2^ (%)	
T vs. C	−0.41	−8.38 to 7.55	0.89	24.13	<0.0001	75.14	Random
TT vs. CC	1.19	−6.07 to 8.47	0.68	24.73	<0.0001	75.44	Random
CT vs. CC	3.00	−2.59 to 8.59	0.22	12.72	0.04	52.86	Random
TT+CT vs. CC	2.03	−4.85 to 8.91	0.48	16.92	0.01	64.54	Random
TT vs. CC+CT	−0.60	−6.22 to 5.00	0.79	19.59	0.003	69.38	Random

### Test of Heterogeneity

Heterogeneity among selected published studies was examined by Q-test and I^2^ statistics and their outcomes have been shown in Table 3. Heterogeneity was observed in all the models, thus random effect model was used for calculation of combined OR and 95% CI [23].

### Meta-analysis Results

**Figure 1:**
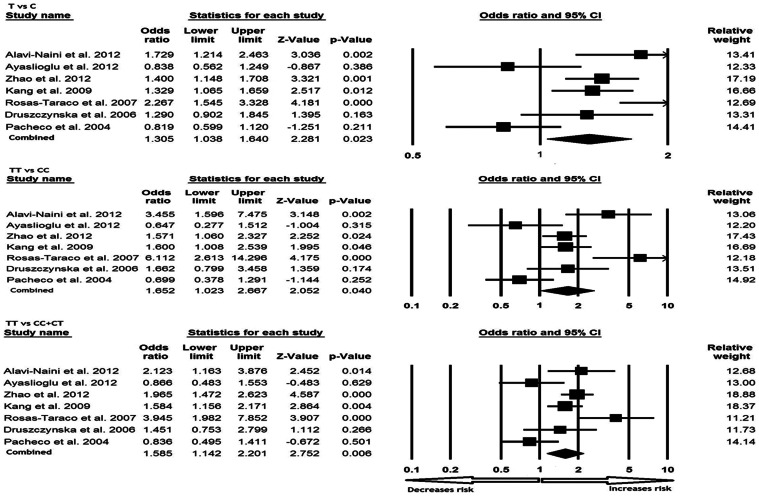
Forest plots (C vs. T; TT vs. CC; TT vs. CC+CT) of CD14 −159 C>T polymorphism in association with TB risk. A meta-analysis was performed including previous reports by comprehensive meta-analysis program. Random model of meta-analysis was employed for calculation of the combined odds ratios and p-values.

**Figure 2:**
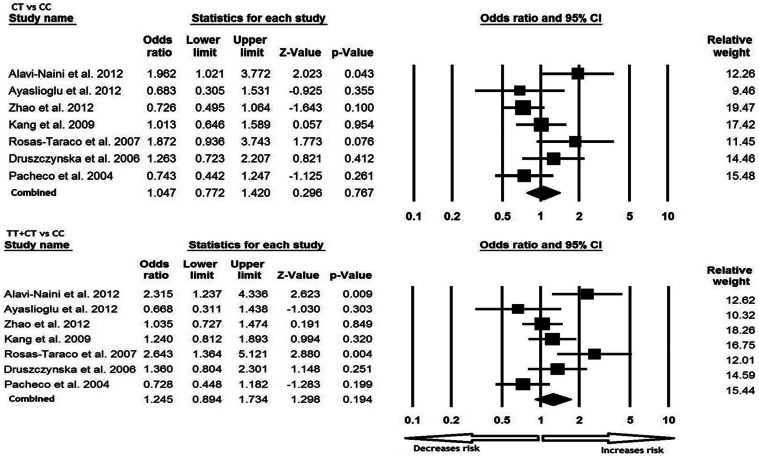
Forest plots (CT vs. CC; TT+CT vs. CC) of CD14 −159 C>T polymorphism in association with TB risk. A meta-analysis was performed including previous reports by comprehensive meta-analysis program. Random model of meta-analysis was employed for calculation of the combined odds ratios and p-values.

All the seven studies were pooled together which resulted into 1389 TB cases and 1421 controls, and meta-analysis was performed using random effects model (based on heterogeneity test) to assess the overall association of CD14 −159 C>T polymorphism with TB risk. The variant T allele demonstrated significant association with the risk of developing TB in terms of frequency in comparison with wild allele (T vs. C: p-value = 0.023; OR = 1.305, 95% CI = 1.038 to 1.640). Similarly, homozygous mutant genotype TT also showed increased risk of TB as compared with the wild type homozygous CC genotype (TT vs. CC; p-value = 0.040; OR = 1.652, 95% CI = 1.023 to 2.667). In addition, analysis of the dominant genetic model revealed 1.5 fold increased risk of developing TB (TT vs. CC+CT: p-value = 0.006; OR = 1.585, 95% CI = 1.142 to 2.201) (Figure 1). Whereas, heterozygous genotype CT (CT vs. CC: p-value = 0.767; OR = 1.047, 95% CI = 0.772 to 1.420) and recessive model (TT+CT vs. CC: p-value = 0.194; OR = 1.245, 95% CI = 0.894 to 1.734) failed to demonstrate significant increase in risk of developing TB as compared to CC genotype (Figure 2).

## Discussion

It is well established that TB susceptibility is determined not only by the infectious agent and environmental factors but also by the host genetic factors [27]. Therefore, a number of candidate genes have been investigated to assess the possible association between modulations of TB risk across different population. In the present study, we performed a meta-analysis to examine the relationship between the −159 C>T variant in the promoter region of the CD14 gene with the risk of TB. The purpose of this study was to summarize the collected data from seven research publications included in this study and assess, whether any association exists between CD14 −159 C>T polymorphism and TB risk or not.

CD14 signaling contributes a part of host response to intracellular bacterial pathogens, such as MTB; and increased sCD14 levels in serum and bronchoalveolar lavage fluid of patients with active TB have been well documented [28], [29], and this enhanced sCD14 level mediates the pathogen-induced cell activation of cells lacking membranous CD14 (mCD14) [30]. In view of the functional relevance of CD14 signaling in MTB, it is conceivable that functional variants in the promoter region of CD14 may influence the pathogenesis of TB. The cellular and subcellular level functional studies have also revealed that CD14 −159 C>T promoter polymorphism influences the binding of the Specificity protein (Sp1 and Sp3) transcription factors that regulate the surface expression of CD14 [12]. Here in this study, individuals with CD14 −159 TT genotype had increased serum sCD14 levels, which increases the inflammatory response [31], [32]. Hence, we can speculate that CD14 −159 C>T polymorphism could therefore be a genetic factor for inter-individual variations in susceptibilities towards TB.

In the present analysis, we found an overall increase in TB risk for the carriers of one or two allele variants as compared to wild allele C and homozygous CC genotype. After stratification into dominant and recessive genetic models, the dominant model (TT vs. CC+CT) showed increased (1.5 fold) risk of TB. However, the influence and exact mechanism of CD14 expression levels on the incidence of TB is still unclear. But it can be predicted that an up-regulation of CD14 expression through MTB might help in pathogenesis of TB by easing immune interactions with mannosylated lipoarabinomannan, which subsequently enhances the production of transforming growth factor (TGF)-β and suppresses the immune response [33]. Furthermore, earlier studies have indicated that CD14 play a critical role in regulation of IgE responses via promotion of Th1 differentiation and suppression of Th2-dependent IgE responses [34], [35]. Pacheco et al. (2004) pioneered to investigate the association between the TB risk and CD14 −159 C>T polymorphism [18], thereafter, many studies have been performed to further evaluate the association in different ethnic groups; however the findings were varying and contradictory. Our meta-analysis suggests significant association of CD14 −159 C>T polymorphism with the increased risk of developing TB. Nevertheless, the etiology of TB is polygenic in nature and a single genetic variant is typically inadequate to forecast the risk of this infectious disease. Also, the major characteristic of CD14 gene −159 C>T polymorphism is that its incidence is varying substantially between different races or ethnicities. In this meta-analysis we found inter-study heterogeneity for CD14 −159 C>T polymorphism, which was probably due to various factors, such as, ethnicity, and allele and genotype distributions for −159 C>T locus. This study has several limitations which need to be addressed in future studies, for e.g., our assessment was based on relatively fewer studies possibly due to the fact that the papers included in our meta-analysis were limited to those published in English only. Further, due to limited number of reports we failed to re-analyze the association of CD14 −159 C>T polymorphism with increase TB risk in different ethnic populations.

### Conclusion

In conclusion, our meta-analysis results suggest that −159 C>T polymorphism in the CD14 promoter could be employed as new risk factor for TB. Future well designed large studies incorporated with environmental factors might be necessary to validate this association in different populations in order to deduce the susceptibility of TB. Such studies might eventually lead to a better and more comprehensive understanding of the association between the CD14 −159 C>T polymorphism and TB risk.

## Supporting Information

Figure S1 **PRISMA 2009 Flow Diagram.**
(DOC)

Checklist S1 **PRISMA 2009 Checklist.**
(DOC)
