# Supplementary material for: CD14 −159 C>T Gene Polymorphism with Increased Risk of Tuberculosis: Evidence from a Meta-Analysis
Source: PLoS One. 2013 May 31;8(5):e64747. doi: 10.1371/journal.pone.0064747 (PMC3669331; doi:10.1371/journal.pone.0064747)
Supplement: Figure S1 — PRISMA 2009 Flow Diagram. (DOC) [file pone.0064747.s001.doc]

**Figure S1 (PRISMA 2009 Flow Diagram)**

**Flow diagram for identifying potential studies for the Meta-analysis**

Relevant studies identified by search strategies: PUBMED, EMBASE **(N=40)**

Studies excluded after reviewing title and abstract (not association study with desired polymorphism, comments and review articles) **(N=31)**

Studies screened for a Meta-analysis **(N=8)**

Studies included in this Meta-analysis with useable information for CD14 -159 C>T **(N=7)**

Study excluded due to (comparing of TB case with Atopy) **(N=1)**
